# Impact of disease burden and late loss of B cell aplasia on the risk of relapse after CD19 chimeric antigen receptor T Cell (Tisagenlecleucel) infusion in pediatric and young adult patients with relapse/refractory acute lymphoblastic leukemia: role of B-cell monitoring

**DOI:** 10.3389/fimmu.2023.1280580

**Published:** 2024-01-16

**Authors:** Águeda Molinos-Quintana, Anna Alonso-Saladrigues, Blanca Herrero, Teresa Caballero-Velázquez, Víctor Galán-Gómez, Melissa Panesso, Montserrat Torrebadell, Javier Delgado-Serrano, Concepción Pérez de Soto, Anna Faura, Berta González-Martínez, Ana Castillo-Robleda, Cristina Diaz-de-Heredia, Antonio Pérez-Martínez, José María Pérez-Hurtado, Susana Rives, José Antonio Pérez-Simón

**Affiliations:** ^1^ Pediatric Unit, Department of Hematology, University Hospital Virgen del Rocío, Instituto de Biomedicina de Sevilla (IBIS)/CSIC, Universidad de Sevilla, Sevilla, Spain; ^2^ CAR T-cell Unit. Leukemia and Lymphoma Department. Pediatric Cancer Center Barcelona (PCCB). Hospital Sant Joan de Déu de Barcelona, Barcelona, Spain; ^3^ Pediatric Hemato-Oncology Department, Peditric University Hospital del Niño Jesús, Madrid, Spain; ^4^ Department of Hematology, University Hospital Virgen del Rocío, Instituto de Biomedicina de Sevilla (IBIS)/CSIC, Universidad de Sevilla, Sevilla, Spain; ^5^ Pediatric Hemato-Oncology Department, University Hospital La Paz, Institute for Health Research (IdiPAZ), Universidad Autónoma de Madrid, Madrid, Spain; ^6^ Division of Pediatric Hematology and Oncology, Hospital Universitari Vall d’Hebron, Vall d’Hebron Research Institute (VHIR), Barcelona, Spain; ^7^ Pediatric Cancer Center Barcelona (PCCB), Institut de Recerca Sant Joan de Déu, Leukemia and Pediatric Hematology Disorders, Developmental Tumors Biology Group, Barcelona, Spain; ^8^ Instituto de Salud Carlos III, Centro de Investigación Biomédica en Red De Enfermedades Raras (CIBERER), Madrid, Spain

**Keywords:** B cell aplasia, late B-cell recovery, pre-infusion tumor burden, CD19 CART-cells, relapsed/refractory acute lymphoblastic leukemia, tisagenlecleucel, B-cell monitoring

## Abstract

**Introduction:**

Loss of B-cell aplasia (BCA) is a well-known marker of functional loss of CD19 CAR-T. Most relapses and loss of BCA occur in the first months after CD19 CAR-T infusion. In addition, high tumor burden (HTB) has shown to have a strong impact on relapse, especially in CD19-negative. However, little is known about the impact of late loss of BCA or the relationship between BCA and pre-infusion tumor burden in patients infused with tisagenlecleucel for relapsed/refractory B-cell acute lymphoblastic leukemia. Therefore, the optimal management of patients with loss of BCA is yet to be defined.

**Methods:**

We conducted a Spanish, multicentre, retrospective study in patients infused with tisagenlecleucel after marketing authorization. A total of 73 consecutively treated patients were evaluated.

**Results:**

Prior to infusion, 39 patients had HTB (≥ 5% bone marrow blasts) whereas 34 had a low tumor burden (LTB) (<5% blasts). Complete remission was achieved in 90.4% of patients, of whom 59% relapsed. HTB was associated with inferior outcomes, with a 12-month EFS of 19.3% compared to 67.2% in patients with LTB (p<0.001) with a median follow-up of 13.5 months (95% CI 12.4 – 16.2). In the HTB subgroup relapses were mainly CD19-negative (72%) whereas in the LTB subgroup they were mainly CD19-positive (71%) (p=0.017). In the LTB group, all CD19-positive relapses were preceded by loss of BCA whereas only 57% (4/7) of HTB patients experienced CD19-positive relapse. We found a positive correlation between loss of BCA and CD19-positive relapse (R-squared: 74) which persisted beyond six months post-infusion. We also explored B-cell recovery over time using two different definitions of loss of BCA and found a few discrepancies. Interestingly, transient immature B-cell recovery followed by BCA was observed in two pediatric patients. In conclusion, HTB has an unfavorable impact on EFS and allo-SCT might be considered in all patients with HTB, regardless of BCA. In patients with LTB, loss of BCA preceded all CD19-positive relapses. CD19-positive relapse was also frequent in patients who lost BCA beyond six months post-infusion. Therefore, these patients are still at significant risk for relapse and close MRD monitoring and/or therapeutic interventions should be considered.

## Introduction

Chimeric antigen receptor (CAR) T-cell therapy has shown promising efficacy for patients with relapsed/refractory B-cell acute lymphoblastic leukemia (R/R B-ALL) (1). Although a high percentage of patients achieve complete remission, a proportion of them will relapse. High leukemic burden pre-infusion and persistence of measurable residual disease (MRD) after tisagenlecleucel infusion are two well-known risk factors for relapse (2, 3). In addition, relapse might be related to loss of functional CAR T-cells or antigen escape (loss of CD19 target) in leukemic cells.

B-cell aplasia (BCA) is a well-known marker of functional CD19 CAR T cells persistence, so B-cell recovery/loss of BCA is associated with the loss of functional CAR T cells. BCA loss mostly occurs during the first year after infusion (4), particularly in the early post infusion phase. In this setting, allogeneic stem cell transplantation (allo-SCT) is highly recommended, when possible (2). Early loss of BCA was established when it occurred within the first six months after CAR T-cell infusion. However, a standard time-frame has not yet been established to separate early from late loss of BCA. Several studies have documented a predictive value of loss of BCA for CD19-positive relapses but not for CD19-negative relapses (4). In addition, the predictive value of loss of BCA could differ depending on tumor burden pre-infusion.

The standardization of BCA monitoring may lead to improved outcomes since it might allow early interventions before relapse takes place. Although there is an international consensus on the grading and management of toxicity (5), there is no consensus on the duration of BCA monitoring. Moreover, inconsistent criteria are used to define BCA. This inconsistency hinders comparison of outcomes from different CAR T-cell constructs or across multicenter studies assessing the impact of BCA on outcomes. Finally, there is scant evidence concerning the real impact of loss of BCA beyond six months after infusion, and clinical decision guidelines are anecdotal. Therefore, the optimal management of patients with loss of BCA is yet to be defined.

We assessed outcomes of long-term BCA monitoring after tisagenlecleucel therapy. Additionally, the impact of loss of BCA was assessed according to pre-infusion leukemic burden and according to its timing (BCA loss before or after six months post-infusion). An evaluation was also performed of B-cell count and immunophenotype of B-cell recovery after CD19 CAR T-cell therapy. We highlight the impact of pre-infusion disease burden on the incidence of relapse, subsequent loss of BCA over time, and event-free survival (EFS) in pediatric/young adults with R/R B-ALL.

## Patients and methods

### Study design

We conducted a Spanish, multicentre, retrospective study from February 2019 to December 2022. The study was composed of 73 children and young adults with R/R B-ALL who received a single intravenous infusion of tisagenlecleucel in five different institutions. All patients met the inclusion criteria of the Spanish state-funded access program, which includes R/R B-ALL after two or more lines of systemic therapy or after transplant. Clinical and laboratory data were collected during routine evaluations and extracted from the GETH-TC (Grupo Español de Trasplante Hematopoyético y Terapia Celular) database and RedCap. All clinical investigation was conducted according to the principles of the Declaration of Helsinki and was approved by the relevant local institutional ethics committee. Informed consent was obtained from all subjects protected by the GETH-TC. Tisagenlecleucel was infused after lymphodepletion chemotherapy (LD) based on fludarabine (30mg/m^2^/day for 4 days) and cyclophosphamide (500 mg/m^2^/day for two days). Bridging therapy was administered at physician´s discretion.

### Definition of response, disease burden and B-cell aplasia assessment

Morphological complete remission (CR) was defined as ≤5% blasts in bone marrow (BM) with complete recovery (CR) or incomplete hematologic recovery (CRi). MRD was evaluated by multiparametric flow cytometry (MFC) performed in the local laboratory. Relapse was defined as any percentage of BM blasts > 0.01% after CR/CRi beyond day 28 (D28) post-infusion or evidence of extramedullary disease. Disease burden was evaluated prior to LD/infusion. High tumor burden (HTB) was defined as >5% of BM blasts by MFC, whereas a count < 5% BM blasts indicated a low tumor burden (LTB).

After infusion, BCA monitoring by MFC was performed in all patients at least once every three months, according to local practice guidelines. We retrospectively collected data on specific peripheral B-cell counts on a per-patient basis. For the monitoring of CD19 CAR-T cells, Human CD19 Protein FITC (Acrobiosystems CD9HF251) or CAR Detection Reagent Biotin (Miltenyi 130-129-550) and Streptavidin PE (BD 405203) were used.

Loss of BCA was defined as first date of reappearance of peripheral B-cells/μL confirmed at two time points (European Bone Marrow Transplantation or EBMT criteria) and/or according to the Pennsylvania criteria established in the pivotal ELIANA phase 2 trial. Hence, loss of BCA was defined as the appearance of >1% of B lymphocytes in peripheral blood lymphocytes or >3% of total white blood cells (or absolute count ≥50/μl) or ≥ 1% of CD19^+^ B-cell population in BM. Ongoing BCA was defined as continuous peripheral B-cell aplasia in the absence of CD19-positive relapse at any site. Transient loss of BCA (tBCA) was established upon transient reappearance of peripheral/bone marrow B-cells not confirmed in subsequent assays, in the absence of CD19-positive relapse at any site. BCA duration was calculated from the day of infusion until confirmation of loss of aplasia (event) or until the last known date of persistence of aplasia.

### Statistical analysis

Event-free survival (EFS) was calculated as the time from infusion to the date of occurrence of relapse, progression or mortality of any cause.

Differences across groups were assessed using Fisher’s exact test or chi-squared test, and Kruskal-Wallis tests for categorical and continuous comparison data, respectively. Kaplan-Meier analysis was used to estimate EFS. EFS was calculated from the date of tisagenlecleucel infusion until the date of relapse, death from any cause, progression after infusion or treatment-related mortality. Where appropriate, EFS was censored to the latest follow-up date or last known alive date without progression. Patients who died before day 28 or did not achieve a CR were included from the EFS analysis. Differences in EFS end points between groups were assessed by the log-rank test. Swimmer plot was created using R software for statistical graphics.

## Results

### Patient characteristics and outcome stream

A total of 73 pediatric-young adult patients with R/R B-ALL received a tisagenlecleucel infusion and were evaluated for ongoing BCA and disease-related outcomes.

Patient´s characteristics are described in **Table 1**. Thirty-three patients (45%) had undergone a previous allo-SCT whereas twelve patients (16%) received inotuzumab as bridging therapy. At the time of LD or pre-infusion, 34 patients had <5% blasts in BM, of whom 15 had negative MRD. Sixteen patients had >50% BM blasts and 23 patients had 5 to 50% BM blasts. Patients were stratified according to the percentage of blasts in BM prior to CAR T-cell infusion: 39 (53.5%) had HTB, whereas 34 (46.5%) were categorized as LTB.

**Table 1:** Characteristics of patients according to pre-infusion tumor burden.

Characteristics	Patients N=73	High disease burden N=39	Low disease burden N=34	P value
Age, years median (Range; percentile p25/75)	10 (1-25; 6/14)	8 (1–22, 5–10)	10,5 (3–25, 8–17)	0,004
Previous allo-SCT n (%)	33 (45)	17 (43.6)	16 (47.1)	0,766
Number of previous therapies, median (range)	2.4	2.4 (0–5)	2.4 (1–5)	
Previous blinatumomab n (%)	5 (7)	3 (7.5)	2 (6)	
Bridging therapy n (%)				0,05
Chemotherapy	61 (84)	36 (93)	25 (73)	
Inotuzumab	12 (16)	3 (7)	9 (26.5)	
Extramedullary disease previous to CAR-T n (%)	17 (23.3)	6^ (15.4)	11^^ (32.3)	0.087
% blasts at peripheral blood median (range)	6.62 (0-94.4)	12.56 (0-94.4)	0	0.005
Fulminant CRS n (%)	2 (2.7)	2 (5)	0	0,49
Progressive disease (< 28 days) n (%)	5 (6.8)	5 (13)	0	0.03
Loss of BCA n (%)	26 (35.6)	5 (19)	21 (81)	0,001
Absolute B-lymphocytes count pre-lymphodepletion mean (CI 95%)	127.3 [51-202]	122 [23.8-221.49]	133.24 [8.5-257]	0,89
Allo-SCT after CAR-T cell infusion n (%)				0,056
No	47 (64)	24 (61.5)	23 (67.6)	
Yes: indicated due to loss of BCA	6 (8.2)	0	6 (17.6)	
Yes, indicated due to relapse	15 (20)	11 (28.2)	4 (11.8)	
Yes, programmed based on a high pre-CAR T cell infusion risk profile*	4 (5.5)	3 (7.7)	1 (2.9)	

Allo-SCT, allogenic stem cell transplantation.

CRS Cytokine-relapse syndrome.

^ 4 central nervous system, 1 testicular and 1 soft tissues.

^^ 7 central nervous system, 3 testicular and 1 parotid.

* Two patients with TP53 mutation and 2 patients with that information not available.

**Figure 1:**
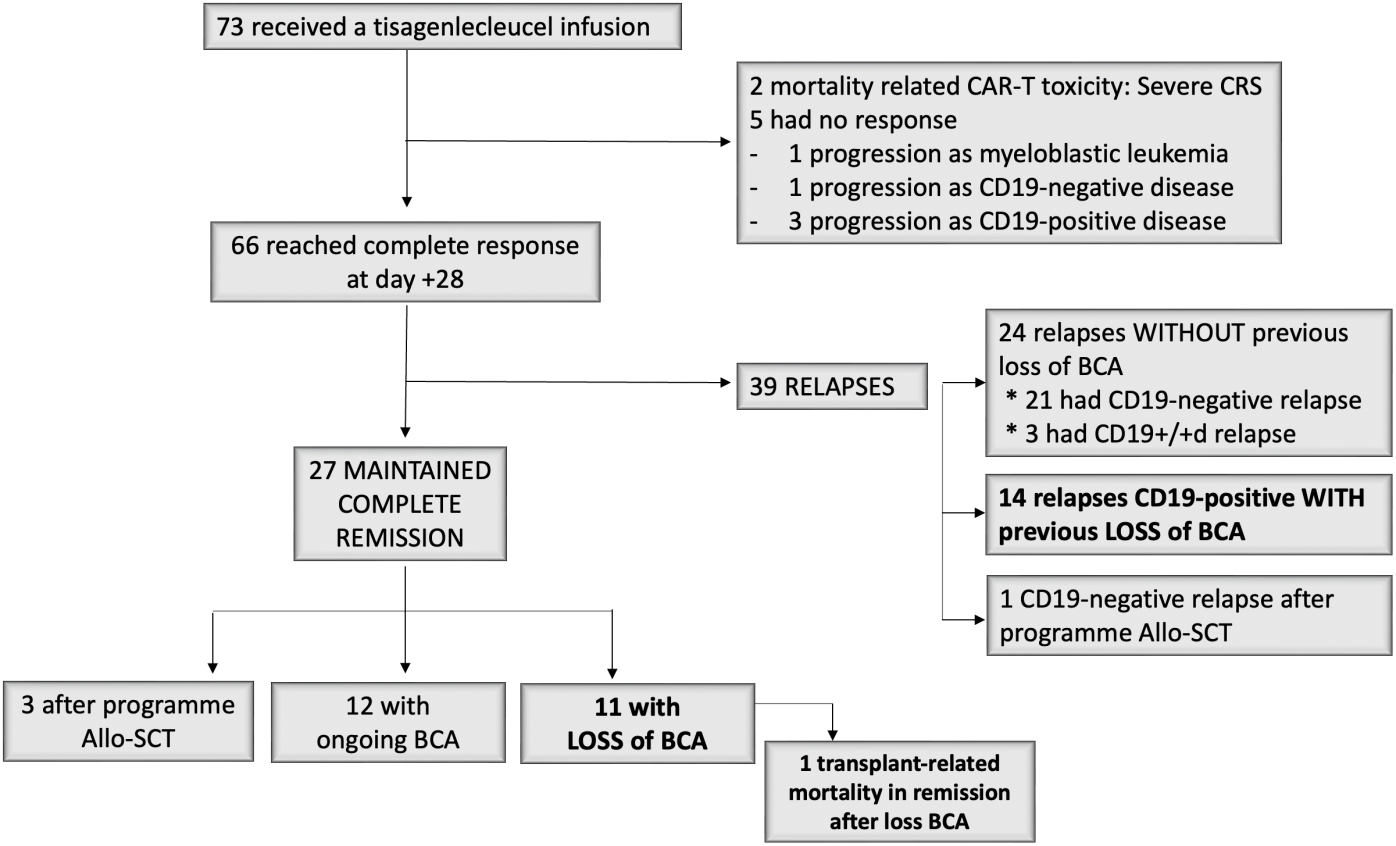
Study profile.

In total, 66 out of the 73 patients (90.4%) achieved CR/CRi at day +28 (D28) after infusion, all of them being in remission with negative MRD by MFC. Among them, a patient had positive molecular MRD (ETV6-RUNX1 positive) at D28; this patient subsequently suffered a CD19-positive relapse three months post-infusion. Two patients (2.7%) died from tisagenlecleucel toxicity prior to CR evaluation at D28. With a median of 13.5 months (95% CI 12.4 – 16.2), 39 patients (59%) relapsed, of which 22 (56.4%) had CD19-negative disease. Overall, 27 patients (41%) maintained the complete remission, 12 (44.4%) with ongoing BCA. In the total series, 26 patients exhibited loss of BCA, 14 of which (54%) suffered CD19-positive relapse, while 11 maintained absolute BCA. A patient died in remission due to transplant-related mortality (**Figure 1**).

### Outcomes depending on tumor burden

**Figure 2:**
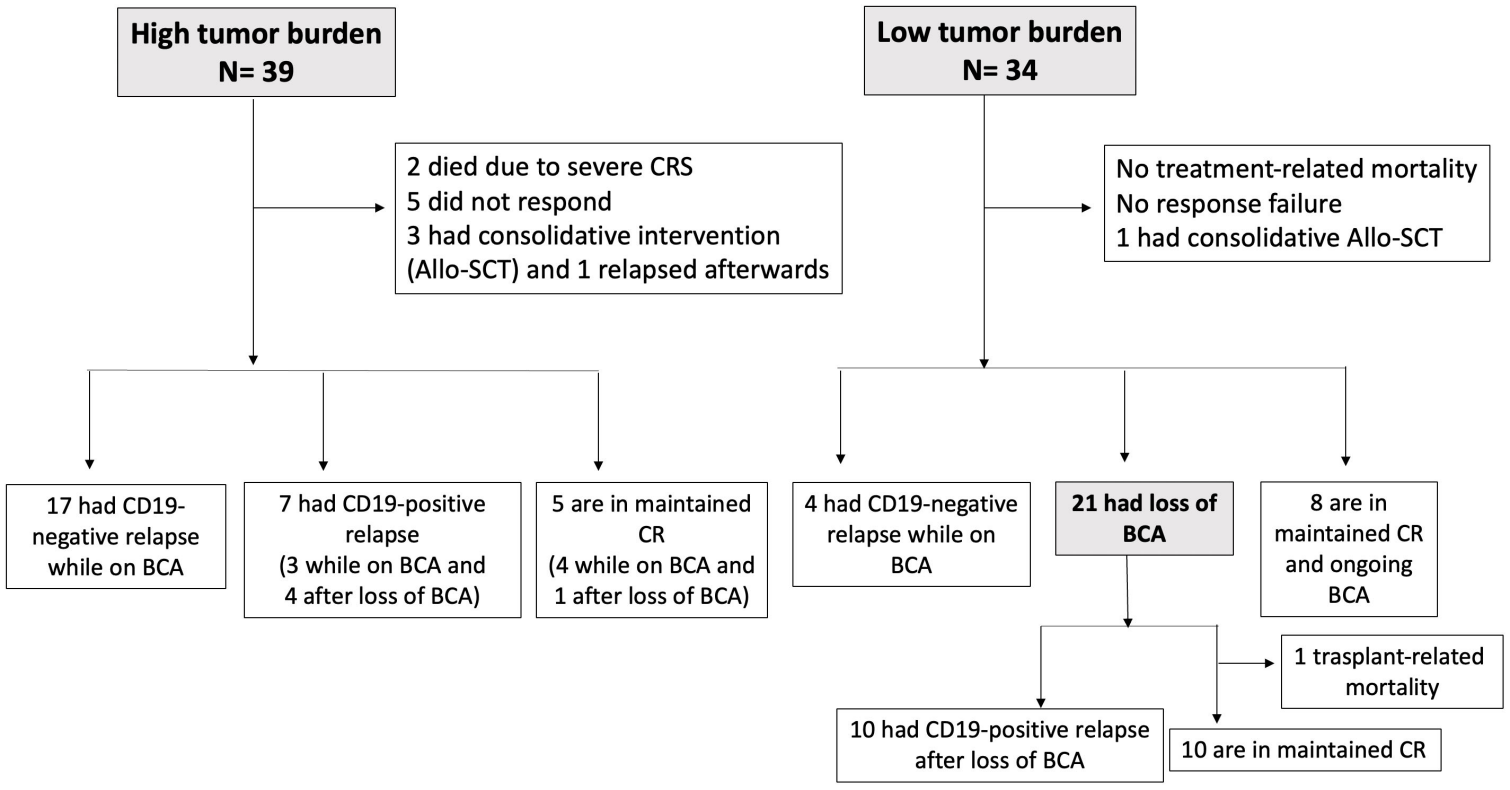
Study profile according to patient´s disease burden pre-infusion.

**Figure 3:**
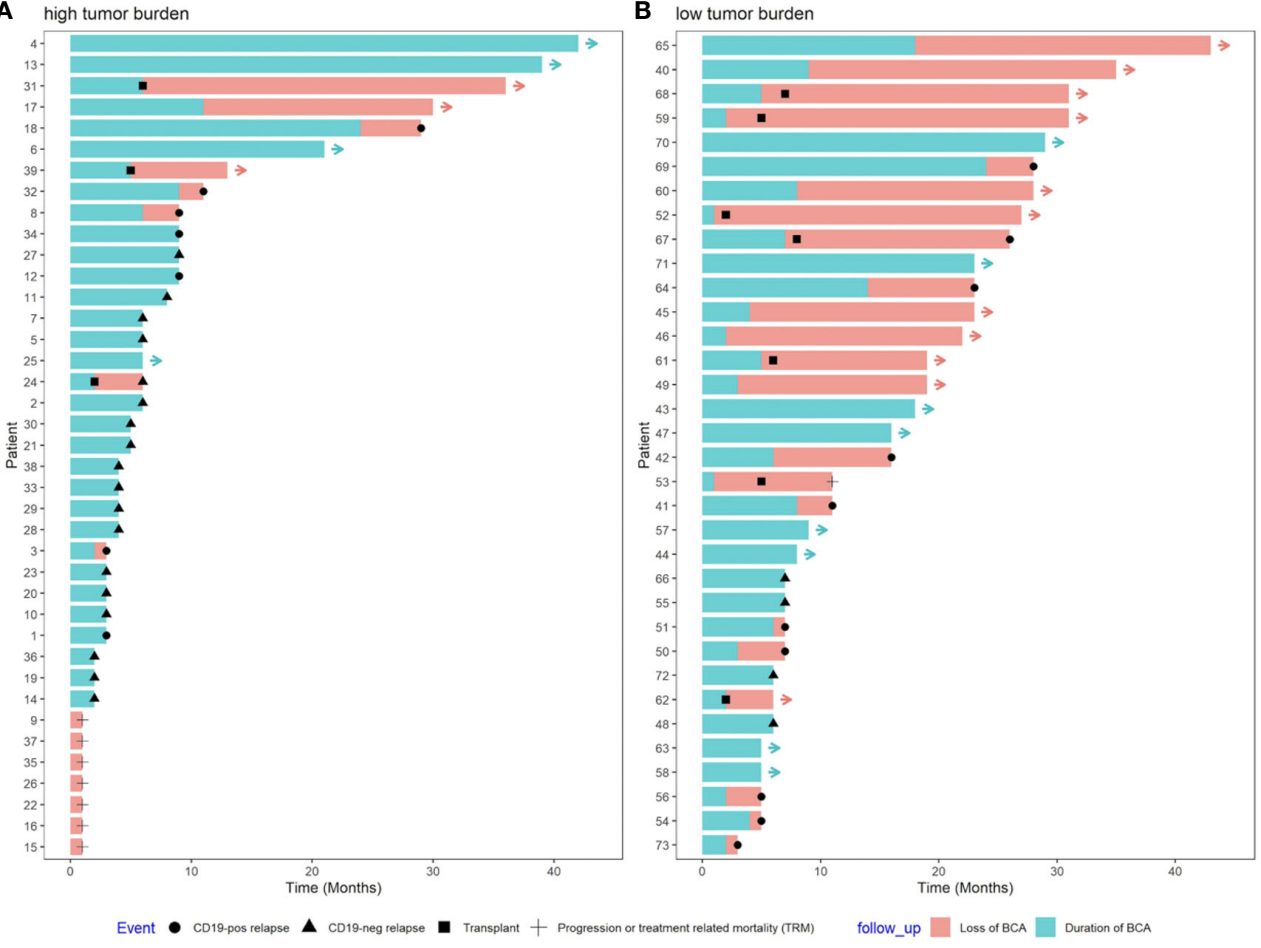
Swimmer plot of **(A)** high tumor burden (HTB) and **(B)** low tumor burden (LTB) disease. HTB patients suffered the vast majority of CD19 negative relapses early after infusion and all the cases of treatment-related mortality. Ten out of 14 relapses in the LTB subset were CD19 positive and all of them were preceded by loss of BCA.

Thirty-nine patients (53.4%) had HTB at the time of infusion; five of them progressed before or at D28 (one with a re-emergence of a CD19-negative clone and three with a CD19-positive clone, whereas the last one exhibited a lineage switch to acute myeloid leukemia). Two patients died because of severe cytokine relapse syndrome (CRS). Seventeen of 32 patients (53.1%) had CD19 negative relapse at a median of three months after infusion (range: 1 to 8 months) while being on BCA. Three patients received a consolidative allo-SCT by physician’s decision while having a MRD negative response with ongoing B-cell aplasia; one of them subsequently had CD19-negative relapse while two patients remained in CR. Seven patients had CD19 positive relapses at a median of 10 months (range 2 to 29), three with ongoing BCA (isolated extramedullary relapse, recurrence of MRD with low CD19-positive expression and isolated BM relapse respectively). Only seven patients in the HTB group (18%) remained in CR at last follow up; four maintained BCA (+6, +21, +39, +42 months); whereas one lost BCA (loss of BCA +11 months), in addition to two patients after allo-SCT (**Figures 2**, **3**).

**Figure 4:**
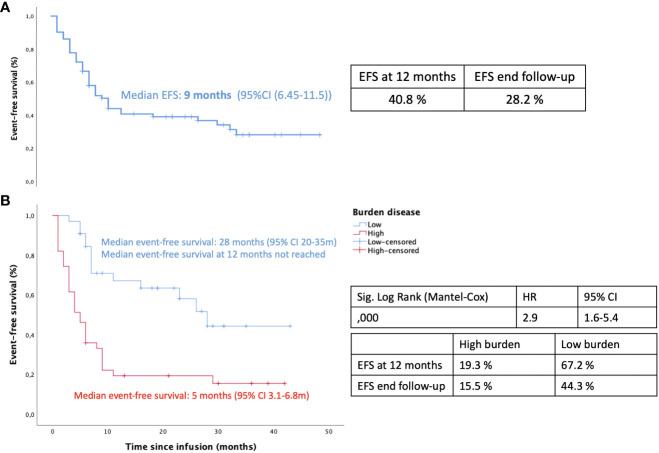
Event free survival: **(A)** Kaplan-Meier curve for the whole series of patients **(B)** Kaplan-Meier analysis for high tumor burden (HTB) vs low tumor burden (LTB).

Thirty-four patients (46.6%) had LTB at the time of infusion, one of whom received a consolidative allo-SCT because of the presence of the TP53 mutation two months after infusion, and continued in CR. Of the remaining 33 LTB patients, 14 relapsed (42%) versus 24 (83%) out of the 29 patients in the HTB cohort (p=0.002), excluding three patients who received a consolidative allo-SCT. Ten out of these 14 relapses in the LTB subgroup were CD19-positive and occurred at a median of 13 months (range 3 to 28) after infusion; all cases were preceded by loss of BCA at a median of six months (range 2-24). Only four relapses were CD19-negative without prior loss of BCA (**Figures 2**, **3**). CD19-negative relapses occurred at a median of 6.5 months (range 6 to 7), as compared to a median of 3 months in the HTB group. Therefore, in the LTB subgroup, only 28% (4/14) of relapses were CD19-negative, as compared to 72% (18/25) in the HTB group (p=0.017). In contrast, 71% (10/14) and 28% (7/25) of relapses were CD19-positive, respectively (p=0.017). Regarding outcomes, with a median follow up of 13.5 months (95% CI 12.4 – 16.2), EFS at 12 months was 40.8% for the total series. More specifically, 12-month EFS was 67.2% and 19.3% for patients with LTB and HTB, respectively (p<0.001), with a median EFS not reached for LTB vs 5 months for HTB, respectively (**Figure 4**).

### Outcomes depending on maintenance of BCA

**Figure 5:**
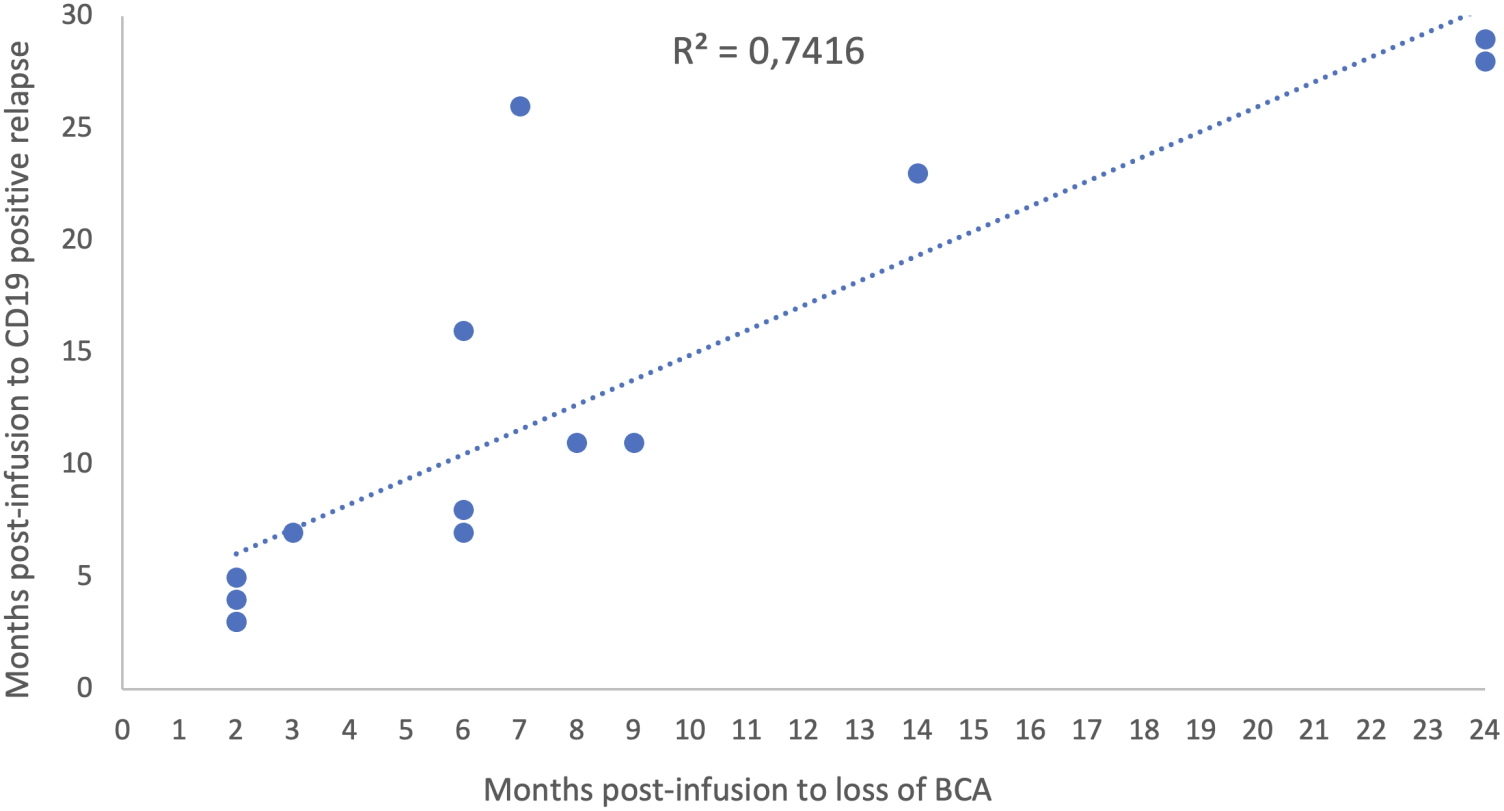
Scatter plot. Relationship between loss of BCA and CD19 positive relapse over time.

Overall, 26 patients showed loss of BCA, 5 in the HTB and 21 in the LTB subgroup. Loss of BCA preceded 100% (10/10) of CD19-positive relapses in the LTB versus only 57% (4/7) in the HTB subgroup. Loss of BCA occurred earlier in the LTB, with a median of 6 months post-infusion (range 1 to 24 months), as compared to 11 months in the five patients with loss of BCA in the HTB group (range 2-27 months). Regarding the available data of CAR-T monitoring by MFC, the median peak of CAR T-cell expansion at a median of 11 days after infusion was lower in the LTB group (65 CAR-T cells/μl, range 3.59-1070), as compared to the HTB group (123 CAR-T cells/μl, range 56-1166) in the 14 and 15 cases analyzed, respectively. In addition, we found a positive correlation between loss of BCA and CD19-positive relapse with a trendline in the scatter plot (R-squared:74%) (**Figure 5**). However, none of the CD19-negative cases of relapse were preceded by loss of BCA.

In the LTB subgroup, relapses occurred in 4 out of 12 patients (33%) while maintained BCA, as compared to 10 out of 21 patients (48%) with prior loss of BCA. Regarding the time of loss of BCA, twelve patients lost BCA within the first six months, five of whom underwent allo-SCT, and only one experienced relapse. Of the remaining seven patients, in which no further intervention was planned, four suffered CD19-positive relapse one to four months after loss of BCA; three did not relapse and remained alive. On another note, nine patients lost BCA ≥ 6 months post-infusion, six of which (67%) subsequently suffered a CD19-positive relapse at a median of 7.5 months at +1, +3, +4, +9, +10 and +19 months, respectively, after loss of BCA, one of them after allo-SCT. Interestingly, the percentage of cases of CD19-positive relapse was nearly doubled (67% vs 36%), although remained non-statistically significant when loss of BCA occurred ≥ 6 months after infusion, as compared to early time-points (p=0.37). Of note, allo-SCT after loss of BCA and prior to relapse was considered in 5 patients with BCA < 6 months after infusion as compared to only one patient with loss of BCA ≥ 6 months after infusion. Clinical interventions after loss of BCA and time from loss of BCA to relapse are summarized in **Table 2**.

**Table 2:** Follow up, outcome and further treatment after loss of BCA or relapse in 21 patients with loss of BCA and LTB; A: early loss of BCA < 6 months, B: late loss of BCA≥ 6 months after infusion and C: 5 patients with HTB.

Table 2A												
	% Blasts pre-LD	Allo-SCT Pre-CART	First absolute B-cell count and %	Months after infusion Absolute loss BCA	Months after infusion Loss BCA Pennsyl	Months after infusion > 1% B-cell at BM	Clinical decision after loss BCA	Relapse (months after infusion)	Duration of remission since loss of BCA (months)	Intervention after relapse	Allo-SCT post CAR-T (Indication)	Last follow up (months after infusion)
1	0	Yes	12 (1,4)	4	4	3	Wait and see	No	Ongoing		No	Alive (21)
2	0	Yes	85 (2)	3	6	3	Wait and see	No	Ongoing		No	Alive (20)
3	2,5	Yes	16 (7,3)	3	3	6	Wait and see	No	Ongoing		No	Alive (18)
4	0	Yes	9 (0,47)	3	5	3	DLI	Yes (7)	4	Inotuzumab and CAR dual	No	Alive with disease (7)
5	0	No	6 (0,4)	1,5	Not reached	1,5	1° Allo-SCT	No	Ongoing		Yes (early loss BCA)	Alive (27)
6	1,3	No	2 (0,12)	1	Not reached	Not reached	1° Allo-SCT	No	Treatment mortality		Yes (early loss BCA)	Death MRT
7	0	No	1 (0,3)	2	Not reached	Not reached	Rapid progression	Yes (4)	2	Inotuzumab and Allo-SCT	Yes (relapse)	Alive (14)
8	0	No	30 (2)	2	2	Not reached	Rapid progression	Yes (5)	3	Inotuzumab and Allo-SCT	Yes (relapse)	Alive (9)
9	1,6	No	175 (3,8)	2	2	3	1° Allo-SCT	No	Ongoing		Yes (early loss BCA)	Alive (29)
10	3,6	No	112 (na)	5	5	5	1° Allo-SCT	No	Ongoing		Yes (early loss BCA)	Alive (18)
11	0,15	No	36 (6)	5	5	6	1° Allo-SCT	No	Ongoing		Yes (early loss BCA)	Alive (38)
12	0,06	NO	240 (30)	2	2	Non available	Rapid progression	Yes (3)	1	Inotuzumab	Progression	Death (progression)

**Table:** 

Table 2B												
	% Blasts pre-LD	Allo-SCT Pre-CART	First absolute B-cell count (%)	Months after infusion Absolute loss BCA	Months after infusion Loss BCA Pennsyl	Months after infusion >1% B-cell at BM	Clinical decision after loss BCA	Relapse (Months after infusion)	Duration of remission since loss of BCA (months)	Intervention after relapse	Allo-SCT post CAR-T (Indication)	Last follow up (months after infusion)
1	0	Yes	9 (0,15)	9	11	9	Wait and see	No	Ongoing		No	Alive (34)
2	0	Yes	168 (20)	8	8	9	Wait and see	Yes (11)	3	Inotuzumab + reinfusion	No	Death (progression)
3	0	Yes	15 (0,5)	6	7	6	Wait and see Asp-PEG	Yes (16)	10	Inotuzumab	No	Alive. Palliative care (16)
4	0,01	No	20 (2)	6	6	Not reached	Wait and see	Yes (7)	1 (MRD relapse)	CART dual + Allo-SCT	Yes (relapse)	Alive (36)
5	0,46	Yes	22 (0,3)	8	8	6	Wait and see	No	Ongoing		No	Alive (27)
6	0	Yes	476 (14)	14	14	9	Wait and see	Yes (23)	9	Reinfusion	No	Alive. Palliative care (46)
7	0,03	Yes	111 (3)	18	18	12	Wait and see	No	Ongoing		No	Alive (42)
8	0,27	No	28 (7)	7	7	6	1° Allo-SCT	Yes (26)	19	Inotuzumab + 2° Allo-SCT	Yes (loss BCA)	Alive. Recent 2° Allo- SCT (26)
9	0,5	No	72 (4)	24	24	24	Wait and see	Yes (28)	4	Chemotherapy Waiting for 1° Allo-SCT	Yes (relapse)	Alive with disease (29)

**Table:** 

Table 2C												
ID	% Blasts pre-LD	Allo-SCT Pre-CAR-T	First absolute B-cell count and %	Months after infusion Absolute loss BCA	Months after infusion Loss BCA Pennsyl	Months after infusion > 1% B-cell at BM	Clinical decision after loss BCA	Relapse (months after infusion)	Duration of remission since loss of BCA (months)	Intervention after relapse	Allo-SCT post CAR-T (Indication)	Last follow- up (months after infusion)
1	74	Yes	399 (9,5%)	2	2	3	2° Allo-SCT but rapid progression	Yes (2,5)	0,5	Inotuzumab + 2° Allo-SCT	Yes (Relapse)	Death (progression)
2	41	Yes	15 (0,23%)	6	8	6	Wait and see	Yes (8)	2	Ruxolitinib and dual CAR T 19/22	No	Alive (8)
3	24	Yes	249 (3,5%)	11	11	12	Wait and see	No	Ongoing		No	Alive (28)
4	30,5	Yes	6 (0,1%)	27	Not reached	24	Wait and see	Yes (29)	2	Inotuzumab + 2° Allo-SCT	Yes (Relapse)	2° Allo-SCT (29)
5	40	Yes	23 (1%)	9	9	9	Wait and see	Yes (11)	2	CART reinfusion, blinatumomab, palliative care	No	Death (progression)

Median of peripheral B-cells as first recovery was 89 lymphocytes/ μL (range 1 to 476).

Regarding the duration of BCA in the subgroup of patients who received inotuzumab as bridging therapy (n=12), the median duration of BCA after infusion was four months (range 2-15) in 10 evaluable patients (one patient was refractory and one underwent a consolidative allo-SCT). This duration contrasts the median duration of BCA of 9.5 months (range 2-42) observed in another bridging therapy (n=52). On the other side, only five patients had received blinatumomab as prior therapy. Two of them were refractory at D28 (one had a CD19-negative and the other CD19-positive relapse) and another patient had a CD19-negative relapse at seven months post-infusion. The other two patients remained in CR at last follow up, one of them with early loss of BCA at three months and the other patient with ongoing BCA at six months of follow-up.

### Monitoring of loss of BCA

We compared the two different criteria for establishing loss of BCA definition (EBMT vs Pennsylvania) in the 26 patients that experienced it.

According to the two criteria, peripheral loss of BCA occurred at the same time (within one month) in 18 out of the 26 patients. Of the remaining eight patients (31%), the loss of BCA occurred 2-3 months later in four patients according to the Pennsylvania criteria for peripheral B-cell count, as compared to the EBMT criteria. The remaining four patients never achieved loss of peripheral BCA according to Pennsylvania criteria; the reason is that two of them underwent a first consolidative allo-SCT and the other two patients relapsed quickly with CD19-positive disease. Remarkably, loss of BCA prior to 3 months post infusion occurred in 7 patients according to EMBT criteria (3 out of 7 not meeting peripheral Pennsylvania criteria); four of them relapsed quickly less than 100 days after losing BCA. The other three patients received a consolidative allo-SCT due to early loss of BCA and two of them continued alive and in remission at 27 and 30 months of follow-up, respectively. The other patient died while in remission due to transplant-related mortality.

**Figure 6:**
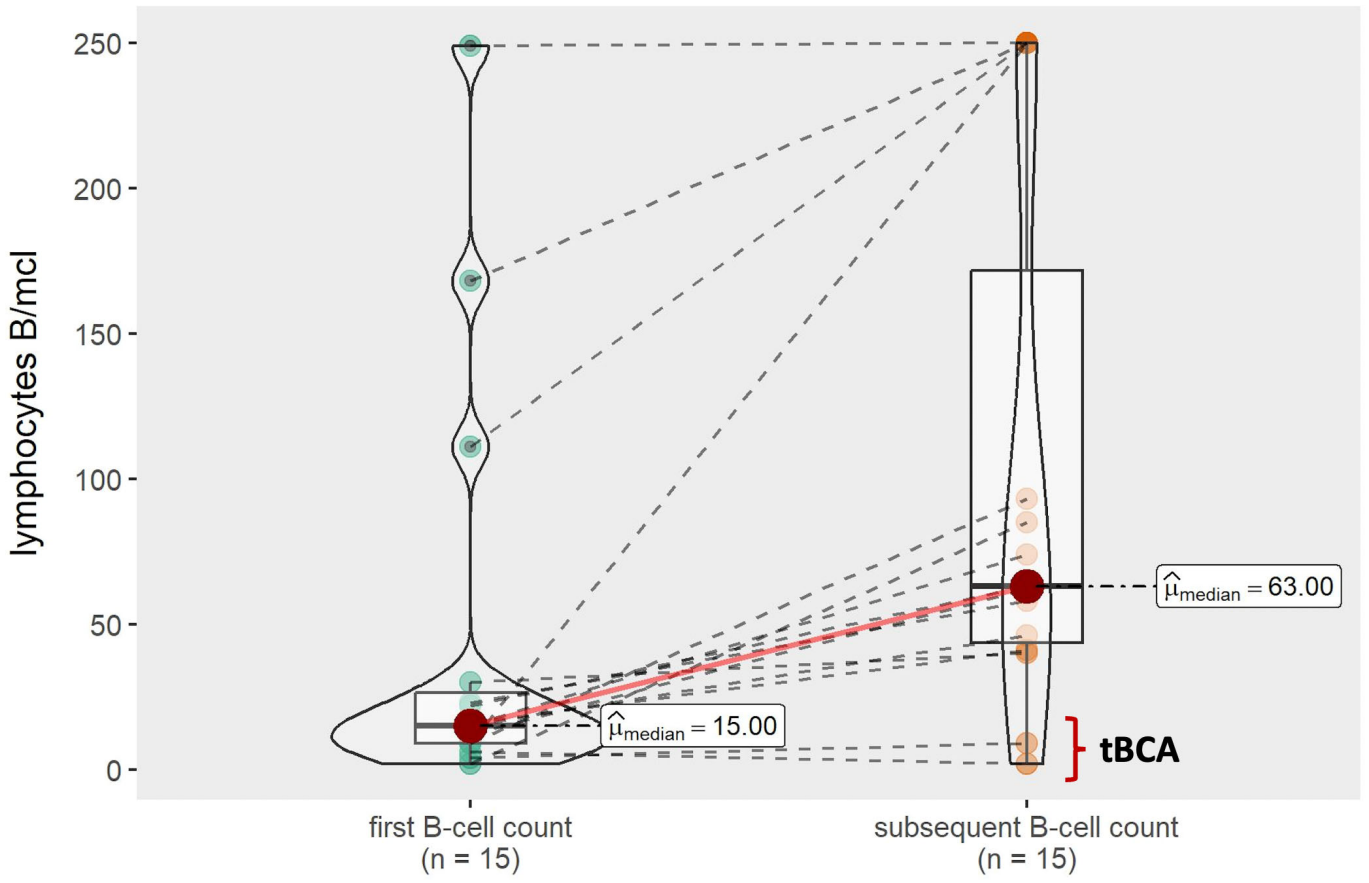
B-cell recovery over time.

**Figure 7:**
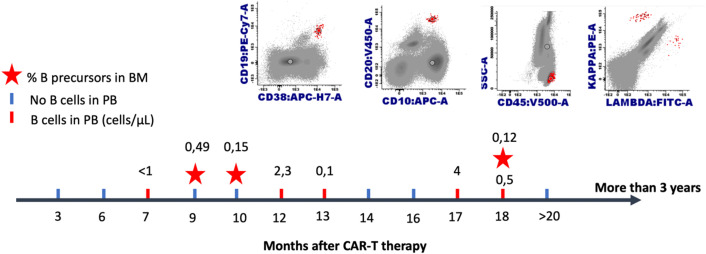
Recovery of transient circulating immature R/R B-ALL pediatric patients after CD19 targeted CAR T cell therapy. Monitoring of a single patient over time: B-lymphocytes in peripheral blood and B precursors in bone marrow.

Excluding patients who underwent allo-SCT or experienced early relapse after loss of BCA (n=13), all other patients with loss of BCA and available subsequent B-cell counts (n=13) tended to show an increase in B-lymphocytes (**Figure 6**). Notably, two additional patients had tBCA with repeated B-cell count < 10 lymphocytes/μl (EBMT criteria) and transient >1% B-cell population in BM (Pennsylvania criteria); these patients subsequently regained absolute BCA in peripheral blood and BM (**Table 3**). Remarkably, the two patients who had transient B-cell recovery had a more immature B-lymphocytes phenotype due to expression of CD10^+^ and CD38^+^ with CD20^+^ surface light chain (Kappa or lambda), as compared to those who eventually lost BCA, who had a more mature B-cell population (CD10^-^, CD38^-/+d^, CD19^+^) (**Figure 7**).

**Table 3:** Follow up of 2 patients with transient BCA.

Months after infusion	Patient 1		Patient 2	
	Absolute peripheral B cells/µL (% in WBC)	% CD19+ B cells in bone marrow	Absolute peripheral B cells/µL (% in WBC)	% CD19+ B cells in bone marrow
1-6	Absolute aplasia	0	Absolute aplasia	0
9	Absolute aplasia	0,49%	Absolute aplasia	0
10	Absolute aplasia	0,15%	Absolute aplasia	
11	*6 (0,1)		*4 (0,3)	
12	*2,3 (0,04)	**3,2%	*9 (0,7)	0,7%
13	0,1 (0,002)		*2 (0,1)	
14			*6 (0,4)	
15	Absolute aplasia		*5 (0,1)	**7,5%
18	0,5 (0,01)	0,12%	Absolute aplasia	**1,4%
24	Absolute aplasia		Absolute aplasia	0,75%
30	Absolute aplasia	0,02%		
Follow-up	3 years Ongoing BCA and CR		2 years Ongoing BCA and CR	

*Loss BCA EBMT criteria **Pennsylvania loss of BCA definition.

## Discussion

In the present study, we report the outcomes and pattern of BCA on a cohort of 73 pediatric/young adult patients with R/R ALL treated with tisagenlecleucel in five centers.

As anticipated, the initial response rate was high, with 90% of evaluable patients experiencing a CR/CRi at D28 after infusion and 100% being negative for MRD by MFC. Unfortunately, relapses were frequent. Leukemic tumor burden significantly correlated with a higher risk for relapse (p=0.006), particularly with CD19-negative relapse, a higher incidence of treatment-related mortality, and a lower EFS, as previously reported (6–11). HTB is considered one of the most important risk factors of relapse, along with MRD (2). In our cohort, more than a half of the relapses (56%) were associated with the loss of CD19 expression. Remarkably, these relapses occurred while on BCA and mainly in the HTB group (82%). Hence, a therapeutic consolidation using pre-emptive consolidative allo-SCT should be strongly considered for this subgroup of patients regardless of BCA status. In our study, few patients with HTB received allo-SCT immediately after tisagenlecleucel treatment. This may be probably due to the encouraging durable response achieved without any additional therapy in the ELIANA trial (4, 12) and also because more than a half of our patients had already received a previous allo-SCT.

Establishing a consensus regarding the best bridging therapy before CAR T-cell infusion remains a challenge, because of multiple individual factors: patient’s condition and disease status, previous toxicity, chemoresistance, and washout period, among others (13). However, our data highlight the poor outcomes in HTB patients. Accordingly, the identification of more effective bridging therapies is urgently needed. The role of other immunotherapies in the setting of CAR T-cell therapy is currently under investigation. Inotuzumab ozogamicin is an anti-CD22 antibody conjugated to calicheamicin used to treat R/R ALL (14). We observed that the majority of our patients treated with inotuzumab achieved LTB pre-infusion (75%). In addition, the median duration of BCA was lower (4 months) in this subset of patients, as previously reported by Krueger et at (15) in a small retrospective cohort of six patients. Thus, the authors reported a median CAR-T persistence of 154 days and a trend towards suboptimal outcomes. It was hypothesized that reduced CD19 antigen stimulation pre-infusion after inotuzumab, could be involved in BCA persistence. These finding should be further explored in the setting of prospective clinical trials (16).

Most centers use BCA as an on-target CAR T-cell effect, thus being a surrogate marker for CAR T-cell activity. However, it is in the LTB group where monitoring for BCA might be most useful, because CD19-negative relapses are much less frequent (11.7% in our series) in LTB patients, as compared to HTB ones (8). On the other hand, as previously reported by Lamble et al. and Pulsipher et al., a percentage of the patients who suffer a CD19-positive relapse do not show a previous loss of BCA (2, 11). These data suggested that BCA could be a suboptimal strategy for predicting not only CD19 negative but also CD19-positive relapses. In addition, CD19-positive lineage chimerism monitored by molecular techniques in patients who received CAR-T19 after allo-SCT, could anticipate relapse earlier than loss of BCA B-cell by MFC (17),

In our study, we describe the pattern of relapse and the role of BCA monitoring according to tumor burden. Using this approach, we observed that only 3 out of 17 (17,6%) CD19-positive relapses occurred while on BCA, as compared to 21-37% in previous studies (2, 11). Importantly, all these relapses while on BCA occurred in the HTB group, while the remaining CD19-positive relapses which occurred in the LTB group were always preceded by loss of BCA. Therefore, BCA monitoring might be optimal only for the LTB subgroup. Notably, as reported by Schultz et al. the loss of BCA preceded relapse in an average of 84 days in the “real world consortium” (18), which is consistent with our median of 105 days in the LTB group. This interval might allow us to intervene prior to the occurrence of a CD19-positive relapse.

Regarding the timing of loss of BCA after CAR T-cell infusion, in the ELIANA trial, the median duration of response for patients with early BCA loss (< 6 months) not undergoing allo-SCT was 12 months. In contrast, response was not reached in the patients who experienced loss of BCA at later time points (12). For this reason, many studies establish six months as the predictive cut-off time-point that identifies patients at a higher or lower risk of relapse (19–21). Accordingly, allo-SCT is recommended when loss of BCA takes place in less than six months. In this regard, Phillips et al. also supported that a duration of BCA < 6 months was a predictor of CD19-positive relapse. Finney et al. documented that, in their experience, all patients with loss of BCA within the first 63 days after infusion relapsed (7). Our data support this idea, since 4 out of 7 patients with early loss of BCA as defined by EBMT criteria, and without any further intervention, relapsed and were treated with salvage therapy and subsequent allo-SCT after relapse. In contrast, for the five patients undergoing allo-SCT after early loss of BCA remained in remission, whereas one died from TRM

Regarding late loss of BCA (> 6 months), we observed that the risk for CD19-positive relapses was still high. In fact, 6 out of 9 patients with loss of BCA ≥ 6 months in the LTB subgroup suffered a subsequent CD19-positive relapse. This finding is consistent with recent reports suggesting that late CD19-positive relapses occur just beyond the first year (11, 22). In the ELIANA trial, there was not a clear plateau in patients treated with tisagenlecleucel, with relapses occurring beyond three years (12, 23). Previous clinical trials and real-world data showing that approximately half of the patients maintain a CAR T-cell mediated remission without any other therapies (18, 24). In our series, watchful waiting was adopted in most patients with late loss of BCA, of which, 67% relapsed. The long-term information available regarding late loss of BCA is limited (2, 7, 25); therefore, monitoring patients with long-term loss of BCA is strongly recommended. The present study confirms a potential window of opportunity for LTB patients with loss of BCA; thus, continued monitoring of LTB patients should be mandatory, irrespective of when the loss of BCA takes place (26). Additionally, close BM MRD monitoring or potential treatment interventions such as maintenance treatment might be offered, as recently suggested by Ghorashian and Gabelli et al. (27, 28).

Based on expert opinions, Buechner et al. (21) proposed that patients with loss of BCA < 3 months should be offered allo-SCT. This algorithm categorizes HTB patients and the loss of BCA within 3-6 months after infusion into the intermediate risk group. The authors recommend close monitoring of MRD, while the decision for or against allo-SCT should be based on the duration of BCA and other potential salvage options on the basis of prior therapies and MRD re-appearance. Recently, Gabelli et al. adopted an oral maintenance chemotherapy strategy with or without monthly pulses of vincristine and dexamethasone in patients with early loss of BCA < 6 months which had a prior transplant, or in absence of a well-matched donor or in case of contraindication of allo-SCT due to co-morbidities (28).

Based on our results, we strongly recommend that HTB patients, irrespective of their BCA status, along with LTB patients with loss of BCA < 3 months should be considered high-risk patients. On another note, LTB patients who experience loss of BCA 3 to 6 months after infusion should be categorized as intermediate-high risk patients. For LTB patients with later loss of BCA, we agree with the strategy of close MRD monitoring and individualized potential salvage options, and/or prior allo-SCT. We encourage the search for novel strategies to prevent relapse when the loss of BCA takes place. These strategies might include maintenance chemotherapy to prevent relapse in previous transplant recipients (28).

Several studies describe a longer persistence of 4-1BB, as compared to CD28-based CAR T-cells. On the other hand, 4-1BB has been associated with a higher resistance to CAR-T cells exhaustion (29). Thus, the role of BCA monitoring might differ across different CAR-T products; therefore, our recommendations apply to B-ALL patients treated with Tisagenlecleucel.

A standard definition of loss of BCA is currently lacking, which hinders cross-study comparisons and the establishment of general recommendations (5, 30). Lymphocyte subpopulations were analyzed to identify B-cell recovery, and absolute B-cell counts were determined. We found no significant differences in outcomes based on the criteria used (EBMT vs Pennsylvania). EMBT criteria were observed to be superior only in one scenario. The appearance of B-lymphocytes in peripheral blood or BM< 3 months did identify an extremely high risk of CD19 relapse (2, 7, 30–32), even when the characteristics of peripheral blood did not meet the Pennsylvania criteria. The percentage of CD19-positive progenitors in BM could be helpful in BCA follow-up, but it is not available on a monthly basis, as compared to peripheral blood counts.

When B-cell monitoring was continued over time, we detected an usual trend towards a progressive increase in the number of B-lymphocytes. Nevertheless, the loss of BCA starting from B-cell count under 10 lymphocytes/μl was not always followed by an increase. Therefore, this is the first study to describe transient B-cell recovery (tBCA). This low B-cell recovery corresponded to an immature population of B-cells by MFC previously described (33). MFC could help us to better understand and define the criteria for loss of BCA and B-cell recovery after CD19 CAR T in the future.

In conclusion, BCA monitoring as a surrogate marker for CAR T-cell activity is optimal only for patients with LTB. Monthly control of B-cell recovery should be recommended in the follow up of these patients. Allo-SCT might be considered in all HTB and LTB patients with loss of BCA < 3–6 months. In LTB patients with loss of BCA > 6 months, close MRD monitoring and individualized strategies should be recommended regardless of the timing of B-cell recovery. High tumor burden pre-infusion is a robust risk factor of relapse and must be avoided by optimizing bridging therapy. Finally, a consensus definition of BCA monitoring would allow us to better compare outcomes across pediatric R/R B-ALL trials, thereby facilitating the development of future international consensus for the management of these patients.

## Data availability statement

The raw data supporting the conclusions of this article will be made available by the authors, without undue reservation.

## Ethics statement

The studies involving humans were approved by CEI de los Hospitales Universitarios Virgen Macarena y Virgen del Rocío. The studies were conducted in accordance with the local legislation and institutional requirements. Written informed consent for participation in this study was provided by the participants’ legal guardians/next of kin.

## Author contributions

ÁM-Q: Data curation, Formal Analysis, Methodology, Writing – original draft, Conceptualization, Investigation. AA-S: Data curation, Writing – original draft, Investigation, Methodology. BH: Data curation, Writing – review & editing. TC-V: Investigation, Writing – review & editing, Methodology. VG-G: Data curation, Writing – review & editing. MP: Data curation, Writing – review & editing. MT: Data curation, Investigation, Writing – review & editing. JD-S: Data curation, Writing – review & editing. CP: Data curation, Methodology, Writing – review & editing. AF: Data curation, Writing – review & editing. BG-M: Data curation, Writing – review & editing. AC-R: Data curation, Writing – review & editing. CD: Methodology, Writing – review & editing. AP-M: Methodology, Supervision, Writing – review & editing. JP-H: Conceptualization, Methodology, Writing – review & editing. SR: Conceptualization, Methodology, Supervision, Writing – review & editing. JP-S: Conceptualization, Methodology, Supervision, Writing – review & editing.
