# Corrigendum: Impact of disease burden and late loss of B cell aplasia on the risk of relapse after CD19 chimeric antigen receptor T Cell (Tisagenlecleucel) infusion in pediatric and young adult patients with relapse/refractory acute lymphoblastic leukemia: role of B-cell monitoring

**DOI:** 10.3389/fimmu.2024.1373852

**Published:** 2024-02-07

**Authors:** Águeda Molinos-Quintana, Anna Alonso-Saladrigues, Blanca Herrero, Teresa Caballero-Velázquez, Víctor Galán-Gómez, Melissa Panesso, Montserrat Torrebadell, Javier Delgado-Serrano, Concepción Pérez de Soto, Anna Faura, Berta González-Martínez, Ana Castillo-Robleda, Cristina Diaz-de-Heredia, Antonio Pérez-Martínez, José María Pérez-Hurtado, Susana Rives, José Antonio Pérez-Simón

**Affiliations:** ^1^ Pediatric Unit, Department of Hematology, University Hospital Virgen del Rocío, Instituto de Biomedicina de Sevilla (IBIS)/CSIC, Universidad de Sevilla, Sevilla, Spain; ^2^ CAR T-cell Unit. Leukemia and Lymphoma Department. Pediatric Cancer Center Barcelona (PCCB). Hospital Sant Joan de Déu de Barcelona, Barcelona, Spain; ^3^ Pediatric Hemato-Oncology Department, Peditric University Hospital del Niño Jesús, Madrid, Spain; ^4^ Department of Hematology, University Hospital Virgen del Rocío, Instituto de Biomedicina de Sevilla (IBIS)/CSIC, Universidad de Sevilla, Sevilla, Spain; ^5^ Pediatric Hemato-Oncology Department, University Hospital La Paz, Institute for Health Research (IdiPAZ), Universidad Autónoma de Madrid, Madrid, Spain; ^6^ Division of Pediatric Hematology and Oncology, Hospital Universitari Vall d’Hebron, Vall d’Hebron Research Institute (VHIR), Barcelona, Spain; ^7^ Pediatric Cancer Center Barcelona (PCCB), Institut de Recerca Sant Joan de Déu, Leukemia and Pediatric Hematology Disorders, Developmental Tumors Biology Group, Barcelona, Spain; ^8^ Instituto de Salud Carlos III, Centro de Investigación Biomédica en Red De Enfermedades Raras (CIBERER), Madrid, Spain

**Keywords:** B cell aplasia, late B-cell recovery, pre-infusion tumor burden, CD19 CART-cells, relapsed/refractory acute lymphoblastic leukemia, tisagenlecleucel, B-cell monitoring

In the published article, there was an error in the author list, and author “on behalf of **Spanish Group for Bone Marrow Transplantation and Cellular therapy group (GETH-TC)”** was erroneously excluded. The corrected author list appears below.

Águeda Molinos-Quintana^1*^, Anna Alonso-Saladrigues^2*^, Blanca Herrero^3^, Teresa Caballero-Velázquez^4^, Víctor Galán-Gómez^5^, Melissa Panesso^6^, Montserrat Torrebadell^2^, Javier Delgado-Serrano^4^, Concepción Pérez de Soto^1^, Anna Faura^2^, Berta González-Martínez^5^, Ana Castillo-Robleda^3^, Cristina Diaz-de-Heredia^6^, Antonio Pérez-Martínez^5^, José María Pérez-Hurtado^1^, Susana Rives^7,8^, José Antonio Pérez-Simón^4^ on behalf of Spanish Group for Bone Marrow Transplantation and Cellular therapy group (GETH-TC)

The authors apologize for this error and state that this does not change the scientific conclusions of the article in any way. The original article has been updated.

